# Gastric fundal heterotopic pancreas mimicking a gastrointestinal stromal tumour (GIST): a case report and a brief review

**DOI:** 10.1186/s13104-016-1995-5

**Published:** 2016-03-22

**Authors:** Duminda Subasinghe, Sivasuriya Sivaganesh, Niranthi Perera, Dharmabandhu N. Samarasekera

**Affiliations:** University Surgical Unit, The National Hospital of Sri Lanka, 28/1, Ishwari Road, Colombo, Sri Lanka; Department of Pathology, Faculty of Medicine, University of Colombo, Colombo, Sri Lanka

**Keywords:** Heterotopic pancreas, Stomach, Endoscopy, Surgery

## Abstract

**Background:**

Heterotopic pancreas is a rare congenital condition characterised by pancreatic tissue lacking vascular or anatomic communication with the normal pancreas. Most cases of ectopic pancreas are asymptomatic. The preoperative diagnosis of this condition is difficult.

**Case presentation:**

A 50-year-old woman presented with dyspeptic symptoms of 4 years duration. Contrast enhanced CT (computed tomography) scan of abdomen suggested a gastrointestinal stromal tumour in the fundus of the stomach. The patient underwent laparoscopy assisted resection and subsequent histology revealed ectopic pancreatic tissue.

**Conclusion:**

Although heterotopic pancreas is a rare lesion diagnosed on histology, it should be considered in the differential diagnosis of gastric mass lesions and in patients presenting with vague upper gastrointestinal symptoms.

## Background

Heterotopic pancreas is a rare developmental anomaly with a reported incidence of 0.55–14 % at autopsy [1], in approximately one in every 500 upper gastrointestinal surgical specimens and in 0.6–13 % of necropsies [2, 3]. Heterotopic pancreas is referred to as ectopic pancreas, aberrant pancreas, and pancreatic rest. Although it was first described in 1727 by Schultz in an ileal diverticulum, the first histological diagnostic confirmation was described by Klob [4, 5] in 1859. It is presence of pancreatic tissue without anatomic or vascular continuity with the normally developed pancreas. Although it is common to occur intra abdominally from anywhere along distal end of the oesophagus to the colon, it has been reported very rarely in extra abdominal sites such as mediastinal cysts, bronchi, lung, umbilicus and brain [6–8]. Intra-abdominal HP lesions commonly known to occur intestines although fallopian tubes, lymph nodes and spleen were rare sites [9]. Out of gastrointestinal lesions, commonest area is upper gastrointestinal tract i.e. stomach (30 %), duodenum (25 %) and jejunum (15 %). At rare instances it can also occur in association with hepatobiliaty organs such as liver, gallbladder, common bile duct, cystic duct [9].

Heterotopic pancreas is usually found incidentally and is generally asymptomatic. However it may become symptomatic when complicated by inflammation, bleeding, obstruction or malignant transformation [10, 11]. The most common heterotopic site is the stomach commonly involving antrum and prepyloric region on the greater curvature or posterior wall [12].

## Case presentation

**Fig. 1:**
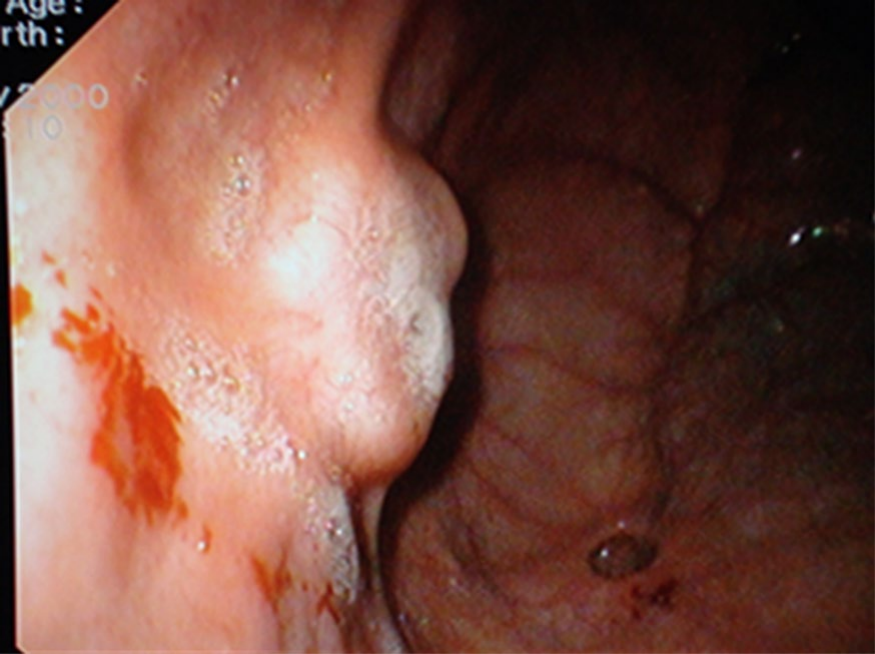
Upper gastrointestinal endoscopy showing gastric fundal mass

**Fig. 2:**
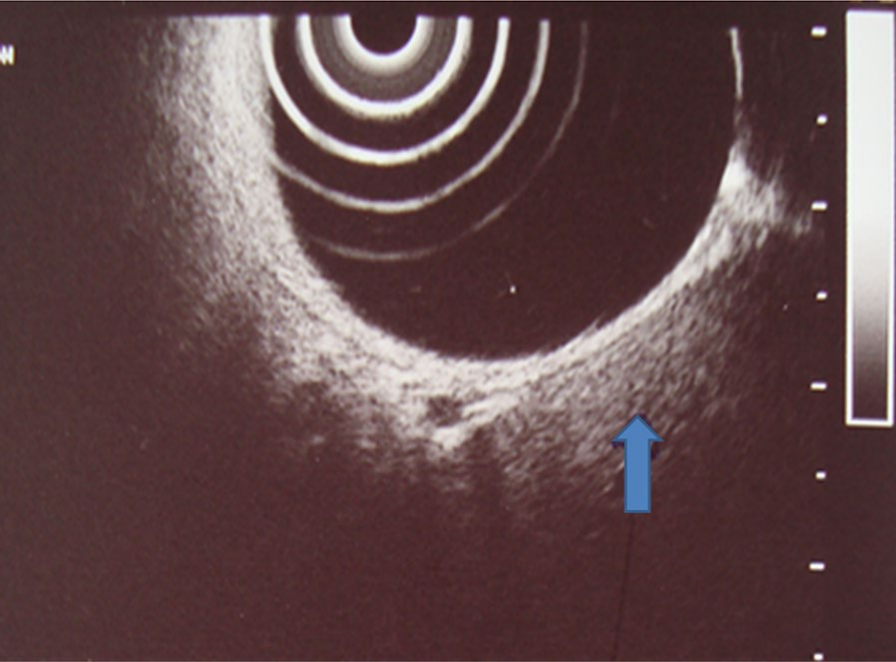
Upper GI endosonography showing a mass lesion in the fundus of stomach

**Fig. 3:**
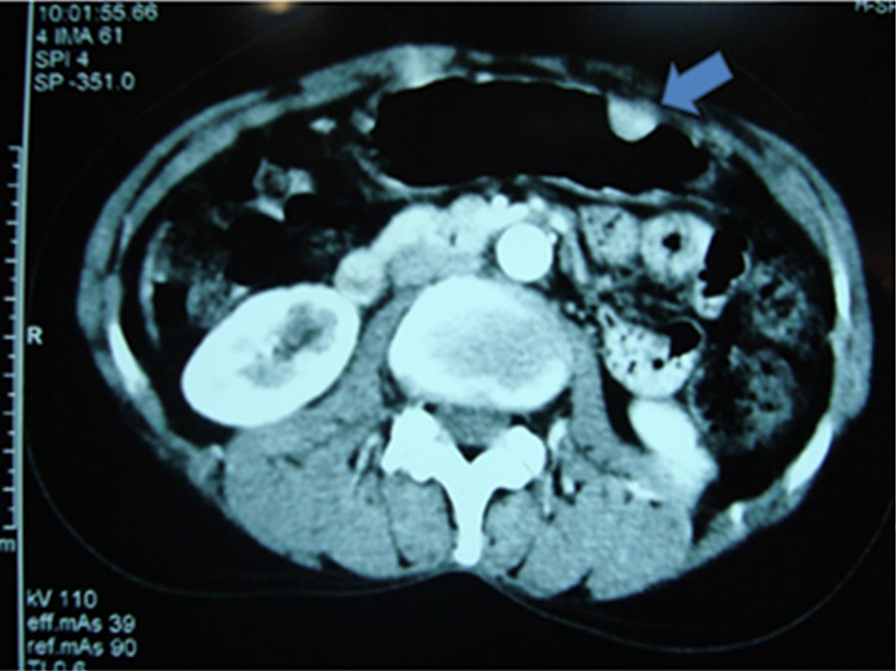
Contrast CT abdomen showing enhancing mass on the anterior gastric wall

**Fig. 4:**
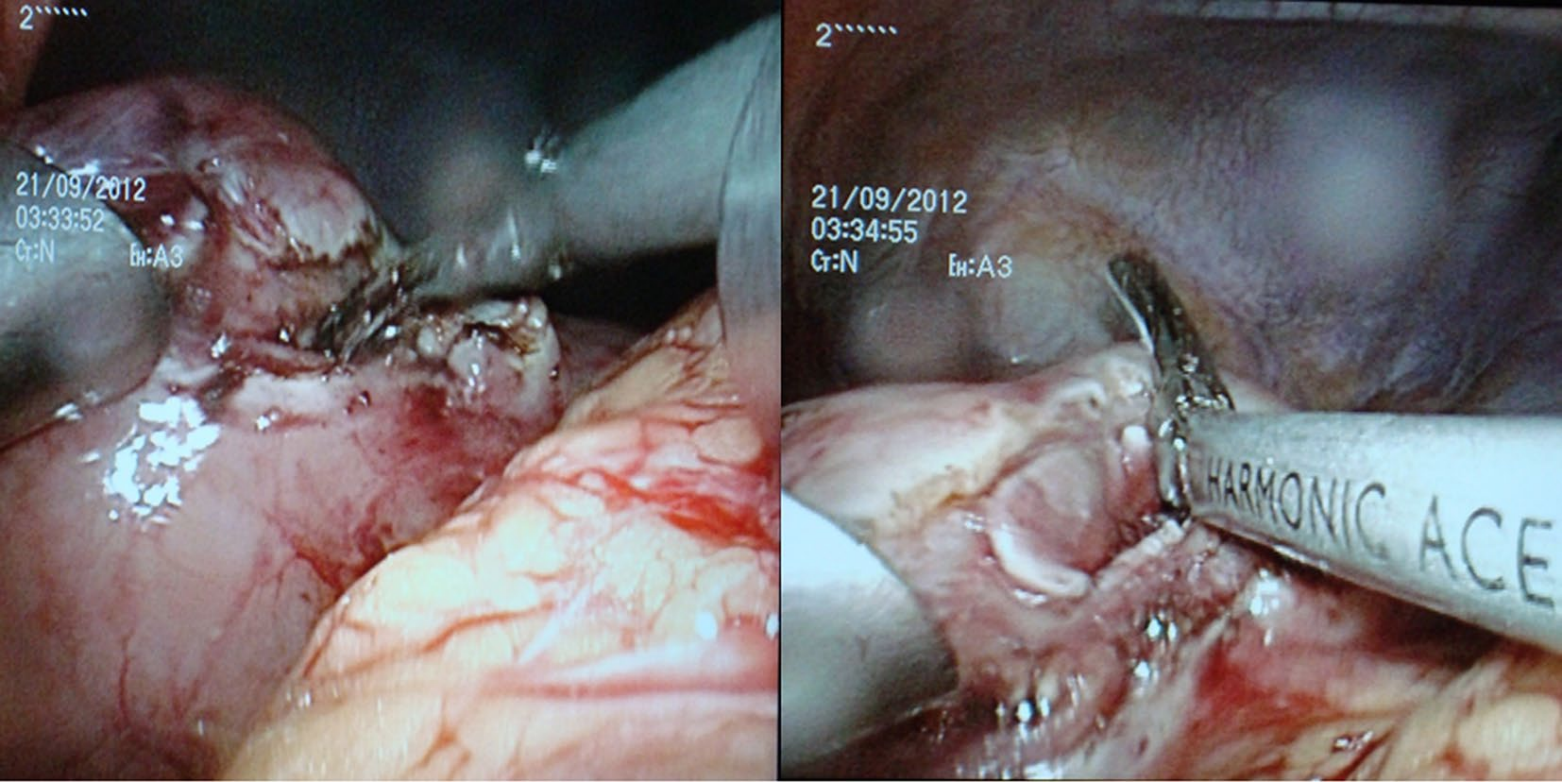
Laparoscopic resection of the gastric fundal mass

**Fig. 5:**
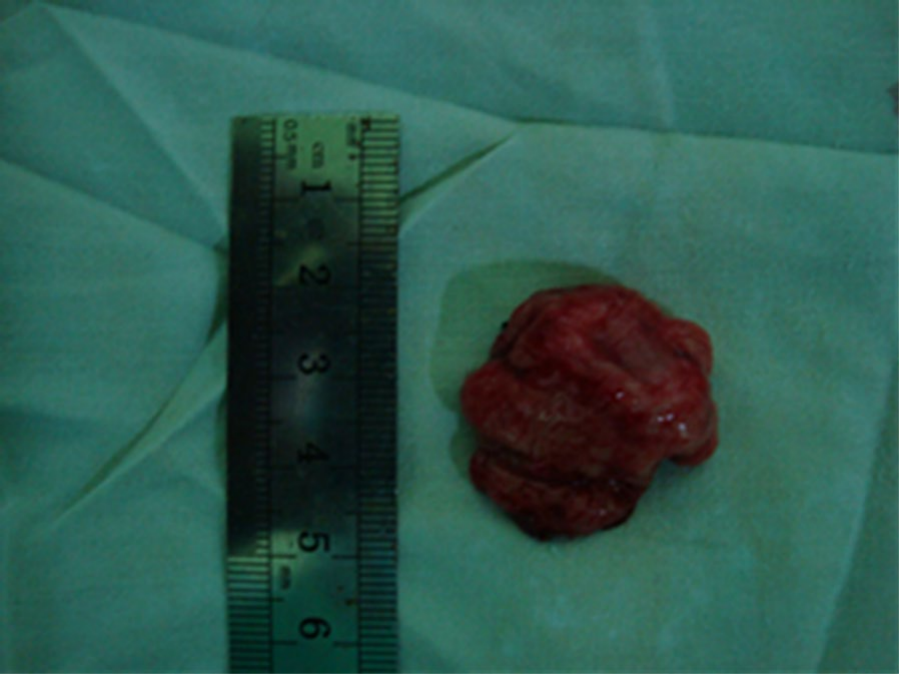
Resected specimen containing tumour

**Fig. 6:**
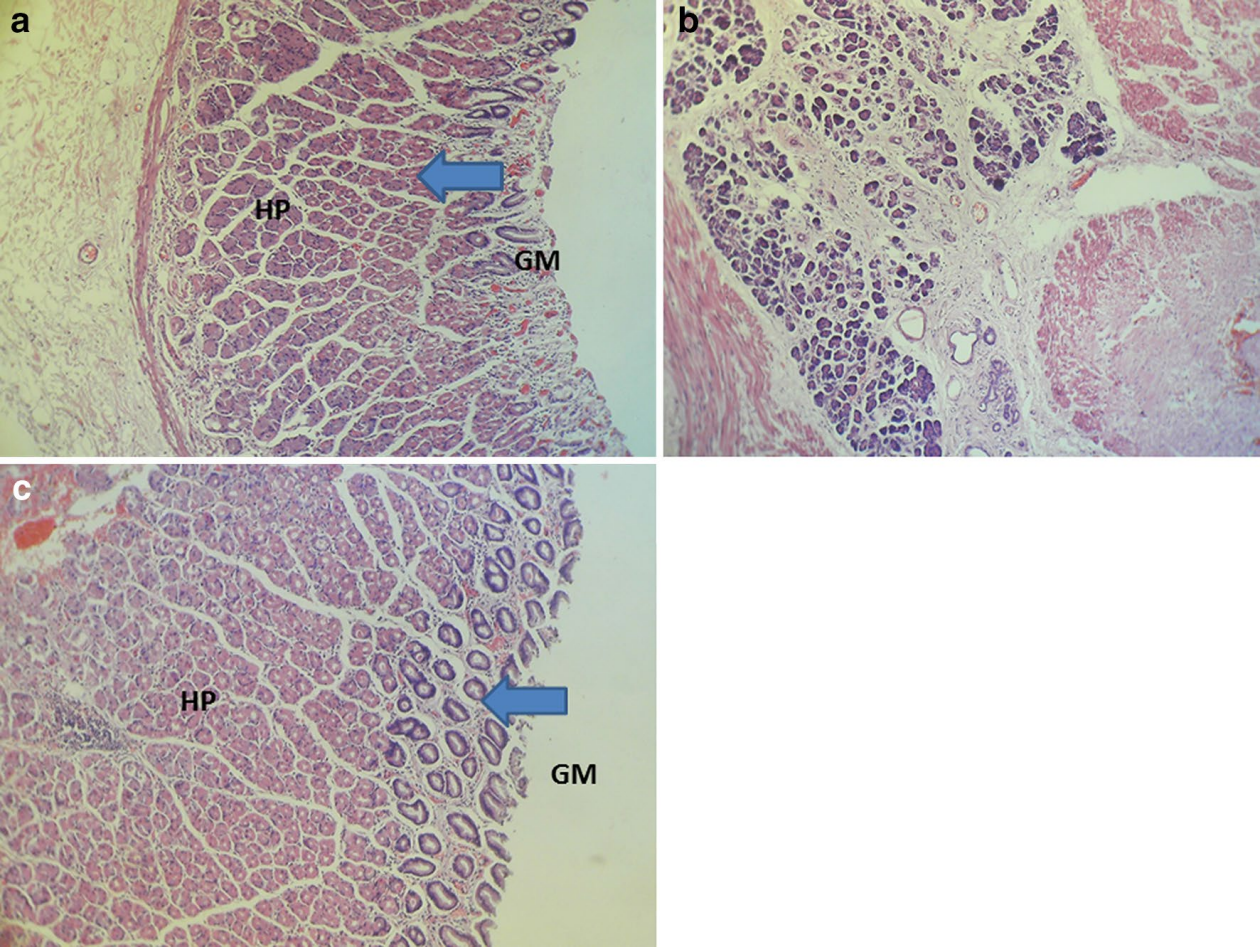
Heterotopic pancreatic tissue (HP) in the submucosa of stomach (**a**, **b**). **c** Heterotopic pancreatic tissue (HP) in the muscularis propria (MP), *GM* Normal gastric mucosa with glands

A 50-year-old woman presented with burning epigastric pain, loss of appetite and associated GORD (gastro-oesophageal reflux disease) symptoms for 4 years duration. She had a history of worsening symptoms of severe dyspeptic symptoms. There was no history of loss of appetite, post prandial vomiting or gastrointestinal bleeding. Previously she had undergone several upper GI (Gastrointestinal) endoscopic examinations at a local hospital for epigastric pain, dyspeptic symptoms and found to have a hyperplastic polyp on biopsy. She gave a history of diabetes mellitus with satisfactory glycaemic control on oral hypoglycaemic drugs. She was on long standing proton pump inhibitors and antacids to relieve symptoms. Physical examination was unremarkable. Her routine laboratory investigations were normal. Upper gastrointestinal endoscopy showed an elevated area of mucosa resembling a sessile polyp in the gastric fundus and an adjacent diverticulum (Fig. 1). On upper GI endosonography (Fig. 2), a solid mass in the region of the gastric fundus was visualized. Contrast enhanced CT scan of abdomen (Fig. 3) showed a well-defined predominantly homogenously enhanced area (Hounsfield 60 unit) on the anterior wall of the stomach in the region of the fundus. Other abdominal organs including pancreas were normal. The operative plan was to perform on table upper GI endoscopy followed by laparoscopic wedge excision of the fundal mass. Diagnostic laparoscopy was normal. There were no regional lymph node enlargement or peritoneal deposits. Other pelvic organs appeared normal on laparoscopy. Laparoscopic surgery (Fig. 4) was converted to open approach due to the bleeding from the short gastric vessels and wide local excision of the mass (1.6 × 1.5 × 1 cm) with a small cuff of the stomach (Fig. 5) was carried out. The post operative period was uneventful and she was discharged on post operative day seven. Histological examination of the specimen (Fig. 6) revealed a heterotopic pancreatic tissue(1 × 2 × 3 cm) in the submucosa and muscularis propria of the stomach. Heterotopic pancreatic tissue comprised of acinar and ductal structures together with scattered islets of langerhan. Her post-operative period was uneventful and she was discharged on 4th post-operative day.

## Discussion

Although several theories have been proposed to explain the pathogenesis, the exact causes of heterotopic pancreas are still not recognized. Embryological basis for heterotopic pancreas is believed to arise during rotation of the foregut, when fragments of the pancreas become separated from the main body and are deposited at ectopic sites [11, 13]. Gastric antrum is the commonest site for heterotopic pancreatic tissues in stomach which accounts about 85–95 %, being more common along the greater curvature. Our patient had the lesion in the anterior wall of stomach near fundus. The symptoms of this entity depend upon the anatomical location and are nonspecific. The diagnosis of ectopic pancreas is difficult despite the development of modern diagnostic methods such as computerized tomography, ultrasonography, and endoscopic ultrasonography because they are not very specific in the diagnosis. Therefore it remains a diagnostic challenge. Endoscopic examination has become useful adjunct in the evaluation of submucosal lesions. The endoscopic picture of heterotopic pancreas usually reveals broad-based, umbilicated, firm, slightly irregular submucosal lesion in the stomach, or elsewhere in the gastrointestinal tract. Although positive biopsy establishes the diagnosis, in most cases, biopsies are superficial and therefore non-diagnostic. The main differential diagnosis for heterotopic pancreatic tissue includes gastrointestinal stromal tumours, gastrointestinal autonomic nerve tumour, gastric carcinoids, lymphoma or gastric carcinoma which can be misinterpreted on imaging studies or endoscopic examinations [14, 15]. There are predictive features on CT such as prominently enhancing overlying mucosa, location, growth pattern, and lesion border which helps in the differentiation of HP tissue from GIST (gastrointestinal stromal tumour) and leiomyoma [16]. Generally it has been suggested that heterotopic pancreas in the stomach is difficult to differentiate from other submucosal tumors on CT as happened in our patient. Therefore symptomatic patients require surgical exploration in order to obtain a definitive diagnosis and to exclude malignancy. Local excision is adequate for benign looking lesions [16]. The management of asymptomatic, incidentally detected HP remains a debate although some evidence suggested in resection of these asymptomatic cases to prevent future complications [4, 5, 11, 12]. The synchronous occurrence of gastrointestinal stromal tumour (GIST) and heterotopic pancreas has been reported once [17]. To our knowledge this is the first case report of a heterotopic pancreatic tissue mimicking a GIST in a patient with diabetes mellitus.

## Conclusions

Heterotopic pancreatic tissue is an incidental rare lesion and gastric fundal site makes it more peculiar and rare. Despite the improvements in the diagnostic endoscopy and cross sectional imaging, it still remains a challenge to differentiate ectopic pancreatic tissue from neoplasms such as GIST. Therefore it prudent to consider it as a differential diagnosis of gastric mass lesions and in patients presented with vague upper gastrointestinal symptoms.

## Consent

We have obtained informed written consent from the patient for publication of this case report and accompanying images.
